# Quantitative Phase Imaging Using Digital Holographic Microscopy Reliably Assesses Morphology and Reflects Elastic Properties of Fibrotic Intestinal Tissue

**DOI:** 10.1038/s41598-019-56045-2

**Published:** 2019-12-18

**Authors:** Arne Bokemeyer, Phil Robin Tepasse, Lena Quill, Philipp Lenz, Emile Rijcken, Michael Vieth, Nik Ding, Steffi Ketelhut, Florian Rieder, Björn Kemper, Dominik Bettenworth

**Affiliations:** 1 Department of Medicine B for Gastroenterology and Hepatology, University Hospital Muenster, Muenster, Germany; 2 Institute of Palliative Care, University Hospital Muenster, Muenster, Germany; 3 Department of General, Visceral and Transplantation Surgery, University Hospital Muenster, Muenster, Germany; 4 Institute of Pathology, Klinikum Bayreuth, University of Erlangen-Nuremberg, Bayreuth, Germany; 5 Department of Medicine, University of Melbourne, Melbourne, Vic Australia; 6 Biomedical Technology Center, University of Muenster, Muenster, Germany; 7 Department of Gastroenterology, Hepatology and Nutrition, Digestive Diseases and Surgery Institute, Cleveland Clinic, Cleveland, Ohio USA

**Keywords:** Biophysics, Crohn's disease, Phase-contrast microscopy

## Abstract

Intestinal strictures are a frequent complication in patients with Crohn’s Disease (CD) and the presence of fibrosis within strictures impacts the therapeutic treatment approach. Here, we evaluate quantitative phase imaging (QPI) using digital holographic microscopy (DHM) for the evaluation of fibrosis within CD strictures. 30 full thickness resection specimens were obtained from non-stenotic and stenotic tissue areas of 15 CD patients. Cryostat sections were analyzed by DHM to measure the spatial distribution of the refractive index (RI) to quantify tissue density. Complementary, histopathological evaluation of H&E staining and immunofluorescence (IF) targeting fibrosis markers served as the gold standard. Moreover, tissue stiffness was evaluated by elastography. RI values assessed by DHM were significantly higher in stenotic compared to non-stenotic tissue areas (p < 0.001). Histopathological analysis using H&E staining and IF confirmed the elevated expression of fibrosis markers in stenotic compared to non-stenotic tissue (all p < 0.001). The RI retrieved by DHM strongly correlated with the amount of fibrosis as determined by IF (p < 0.001; R^2^ = 0.48). Furthermore, elastography detected a significantly higher tissue stiffness in stenotic as compared to non-stenotic tissue sections (p < 0.001). In conclusion, QPI using DHM accurately assesses fibrotic properties of CD-associated strictures and may improve the characterization of CD strictures.

## Introduction

Crohn’s disease (CD) is a common inflammatory disorder of the gastrointestinal tract which is characterized by a discontinuous chronic relapsing disease course^1^. Up to 10% of patients have intestinal strictures at initial diagnosis and over time approximately one third of all CD patients will develop strictures^2,3^. Clinical symptoms of affected patients include obstructive symptoms like abdominal pain, bloating, nausea and vomiting^4^. Intestinal strictures most commonly consist of inflammatory and fibrotic tissue components^4^. The differentiation between fibrosis and inflammation is crucial for the choice of therapy: while anti-inflammatory medication is routinely commenced in CD patients with a predominant inflammatory stricture, predominant fibrotic strictures are treated through endoscopic dilation as well as stricturoplasty or intestinal resection^5–7^. Despite all available diagnostic tools such as cross-sectional imaging including ultrasound (US), computed tomography (CT) and magnetic resonance imaging (MRI), the determination of the exact degree of fibrotic alterations within CD strictures before surgery remains challenging to adapt the therapy accordingly^5^.

Quantitative phase imaging (QPI)^8^ including digital holographic microscopy (DHM)^9^ has been previously used in a large variety of set-ups ranging from single cell studies for the analysis of blood^10^, endothelial^11^ and neuronal cells^12^, for cancer cell research^13^, infections^14^ and cell culture quality control^15,16^ up to the histopathological analysis of tissue samples^17–19^. The operation principle of DHM is based on the detection of the optical path-length delay (OPLD) caused by a mainly transparent unstained specimen against the surrounding tissue. The OPLD can be used for the assessment of the refractive index (RI), which directly correlates to tissue density^18,20,21^. Recently, DHM was used to accurately characterize inflammatory bowel disease-(IBD)-mediated tissue alterations and wound healing processes *in vitro* and was furthermore successfully applied for the histopathological quantification of intestinal inflammation in IBD patients^18,19,22^.

Considering the limited ability of current imaging diagnostics to assess the degree of intestinal fibrosis, this study aimed to evaluate QPI provided by DHM for the determination of fibrosis within CD-associated intestinal strictures.

## Results

### Study population

**Figure 1:**
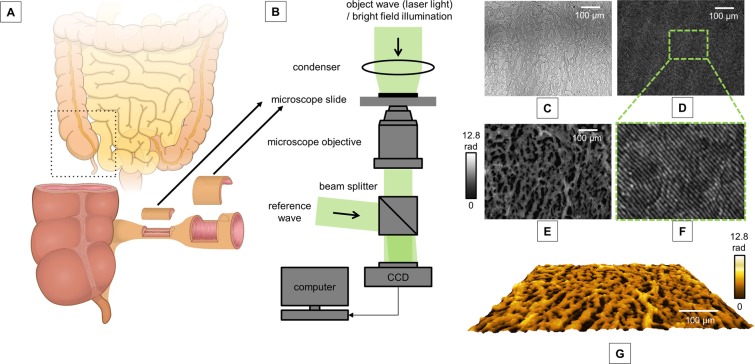
Experimental set-up. (**A**) Full thickness surgical resection specimen of Crohn’s disease patients with a stricturing disease phenotype were obtained from the stenotic segment and the adjacent, non-stenotic segment of the intestinal wall. (**B**) Experimental setup for off-axis digital holographic microscopy (DHM) and bright field imaging; (**C**) Bright field image of representative stenotic tissue; (**D**) corresponding digital off-axis hologram; (**E**) quantitative phase image reconstructed from the digital hologram in **D**; (**F**) enlarged part of the digital hologram that illustrates the off-axis carrier fringes; (**G**) false color coded pseudo 3D representation of the quantitative phase image in **E**.

We evaluated 30 full thickness surgical resection specimen obtained from 15 CD patients. From each patient, one tissue sample was obtained directly from the stricture and another sample was retrieved from the adjacently localized non-stenotic bowel segment (Fig. 1, Table 1). 60% of the patients were female and 40% of the patients were male. The mean age was 43.5 (standard error of mean [SEM]: ± 3.3 years). Patients had a long disease course with a mean duration of 10.0 ± 2.4 years. Prior to surgery, 86.7% and 20.0% of patients suffered from abdominal pain and diarrhea, respectively. The disease activity assessed by the *Crohn’s disease activity index (CDAI)* was 196.6 ± 22.8 points and C-reactive protein was 3.9 ± 1.3 mg/dl. The mean time from initial stricture diagnosis to surgery was 5.4 ± 1.2 months. Most of the patients were being treated by ileocaecal resection (60%) including right hemicolectomy in 13.3% of patients, followed by (sub-) total colectomy including ileocaecal resection (20%), anastomotic resection after a previous ileocaecal resection (13.3%) and left hemicolectomy (6.7%). The mean length of the resected intestinal stricture was 11.2 ± 2.6 cm. 53.3% of our patients were treated with a combination of anti-inflammatory medication prior surgical resection, followed by 33.3% with a monotherapy and 13.3% with no medical therapy. In detail, 40% of all patients received biologics (83.3% anti-tumor-necrosis-α-antibodies and 16.7% Ustekinumab) and/or 40% corticosteroids (83.3% systemic and 16.7% topical), followed by 33.3% receiving azathioprine and 6.7% receiving mesalamine (Table 1).

**Table 1:** Characteristics of patients with Crohn’s disease associated intestinal strictures undergoing surgical resection of the stricture.

Variables	Patients (n = 15)
Age (in years)	43.5 ± 3.3
Female (%)	9 (60.0)
Male (%)	6 (40.0)
Duration of disease (in years)	10.0 ± 2.4
**Clinical symptoms prior surgical resection**	
Abdominal pain (%)	13 (86.7)
Diarrhea (%)	3 (20.0)
Crohn’s Disease activity index	196.6 ± 22.8
**Laboratory parameters**	
White blood cell count (1/l)	10.7 ± 0.9
C-reactive protein (mg/dl)	3.9 ± 1.3
Time from initial diagnosis to surgical resection (months)	5.4 ± 1.2
**Kind of surgical resection (%)**	
Ileocaecal resection	9 (60.0)
Including right hemicolectomy	2 (13.3)
(Sub-) total colectomy including ileocaecal resection	3 (20.0)
Anastomotic resection after ileocaecal resection	2 (13.3)
Left hemicolectomy	1 (6.7)
Length of intestinal stricture (cm)	11.2 ± 2.6
**Medical treatment**	
Biologics	6 (40.0)
Anti-Tumor-Necrosis-Factor-α (anti-TNF-α)	5 (33.3)
Ustekinumab	1 (6.7)
Immunosuppressants	5 (33.3)
Azathioprine	5 (33.3)
Corticosteroids	6 (40.0)
Systemic	5 (33.3)
Topical	1 (6.7)
Mesalamine	1 (6.7)
**Kind of medical therapy**	
Monotherapy	8 (53.3)
Combination therapy	5 (33.3)
No medical therapy	2 (13.3)

Arithmetic mean and standard error of mean (SEM) are reported for continuous variables, and frequencies and percentages are reported for categorical variables.

### Assessment of the optical path length delay and the refractive index of non-stenotic and stenotic intestinal tissue using digital holographic microscopy

**Figure 2:**
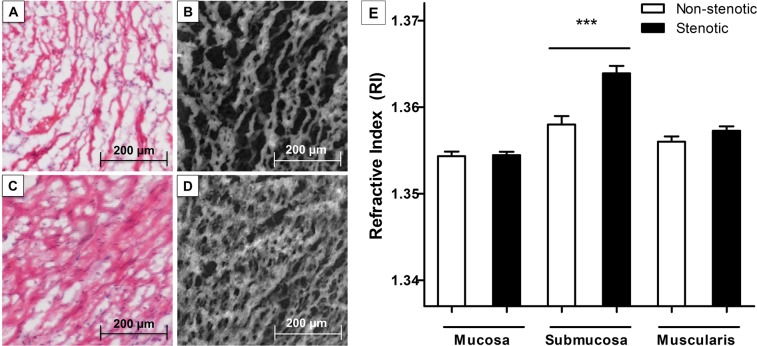
Determination of the refractive index of stenotic and non-stenotic intestinal tissue of Crohn’s disease patients using digital holographic microscopy (DHM). 30 surgical resection specimen of stenotic and non-stenotic intestinal tissue of 15 Crohn’s disease patients were histopathologically evaluated using Hematoxylin-Eosin-(HE)-staining and DHM. Histological evaluation of HE-stainings and the corresponding quantitative DHM phase contrast image (coded to 256 gray levels) revealed marked fibrotic changes of the submucosal layer of stenotic (**C,D**) compared to non-stenotic bowel tissue (**A,B**). The refractive index, determined by DHM, was assessed in all layers of the bowel wall (mucosa, submucosa and muscularis propria) and was significantly elevated in the submucosa of stenotic compared to non-stenotic tissue (p < 0.001). Data are mean ± standard error of mean (SEM). Statistical analysis was performed using Mann-Whitney U test. Two-sided p values < 0.05 were considered statistically significant.

Conventional histological evaluation of Hematoxylin and Eosin-(H&E)-staining of the investigated surgical resection specimen confirmed the presence of non-stenotic and stenotic tissue samples considering changes of the crypt architecture and the presence of submucosal fibrosis (Fig. 2A/C).

QPI using DHM reliably allowed the differentiation of non-stenotic and stenotic tissue samples (Fig. 2B/D/E). The average RI of stenotic tissue was significantly higher compared to non-stenotic tissue (1.359 ± 0.001 vs. 1.356 ± 0.000; p < 0.001; Table 2). Differentiating by bowel wall layer, the RI as assessed using the submucosa of stenotic tissue samples, was significantly increased compared to non-stenotic tissue samples (1.364 ± 0.001 vs. 1.358 ± 0.001; p < 0.001, Table 2 and Fig. 2). The RI of the muscularis propria only tended to be higher in stenotic compared to non-stenotic bowel tissue but did not reach statistical significance (1.357 ± 0.001 vs. 1.356 ± 0.001 [p_muscularis_ = 0.337]; Table 2 and Fig. 2**)** and the RI of the mucosa was similar in stenotic and non-stenotic bowel tissue (1.354 ± 0.000 vs. 1.354 ± 0.001 [p_mucosa_ = 0.833]).

**Table 2:** Significantly prolonged average optical path length delay Δφ and refractive index (RI) in stenotic compared to non-stenotic intestinal tissue of Crohn’s disease patients (surgical resection specimen n = 30; number of patients = 15).

	Non-stenotic tissue (n = 15)		Stenotic tissue (n = 15)		
	Δφ (rad)	RI	Δφ (rad)	RI	P value
Overall	1.544 ± 0.034	1.356 ± 0.000	1.802 ± 0.045	1.359 ± 0.001	<0.001***
Mucosa	1.434 ± 0.044	1.354 ± 0.001	1.445 ± 0.035	1.354 ± 0.000	0.833
Submucosa	1.743 ± 0.080	1.358 ± 0.001	2.228 ± 0.072	1.364 ± 0.001	<0.001***
Muscularis	1.571 ± 0.050	1.356 ± 0.001	1.678 ± 0.043	1.357 ± 0.001	0.337

Arithmetic mean and standard error of mean (SEM) are reported for continuous variables. Statistical analysis was performed using Mann-Whitney U test. Two-sided p values < 0.05 were considered statistically significant.

Most tissue samples were obtained from the ileum (20/30; 67%) and only the minority of tissue samples were obtained from the colon (10/30; 33%). Overall, the average RI measured within ileal or colonic tissue samples was comparable (1.357 ± 0.000 vs. 1.358 ± 0.001; p = 0.181). More specifically, RI values of stenotic and non-stenotic tissue samples were determined separately for ileal and colonic tissue samples and separately for each bowel wall layer: in detail, a significantly elevated RI (accounting for a higher tissue density) was observed in stenotic tissue samples of the submucosa compared to non-stenotic tissue samples in both, ileal and colonic tissue (Ileum: RI_non-stenotic_ 1.357 ± 0.001 vs. RI_stenotic_ 1.363 ± 0.001, p_Ileum_ = 0.004; Colon: RI_non-stenotic_ 1.360 ± 0.001 vs. RI_stenotic_ 1.365 ± 0.001, p_Colon_ = 0.01; Supplementary Information). Furthermore, in ileal tissue, the RI of the muscularis propria in stenotic tissue was significantly increased (RI_non-stenotic_ 1.355 ± 0.001 vs. RI_stenotic_ 1.358 ± 0.001, p = 0.017), while the RI of stenotic tissue layers of the mucosa was only slightly, but not significantly increased (p = 0.585). In colonic tissue, the RI was slightly, but not significantly increased in the mucosa and the muscularis propria (p = 0.505 and p = 0.176) of non-stenotic tissue (however, these colonic tissue results might be interpreted with cautious due to the limited number of available colonic tissue samples).

In addition, the average RI of all patients receiving no medical therapeutics before surgery (n = 2) was compared to the RI of patients receiving medication (n = 13) resulting in no statistically significant difference between both groups (RI_medication-group_ 1.357 ± 0.001 vs. RI_no-medication-group_ 1.356 ± 0.001; p = 0.297).

### Evaluation of fibrotic alterations within tissue samples by immunofluorescence

**Figure 3:**
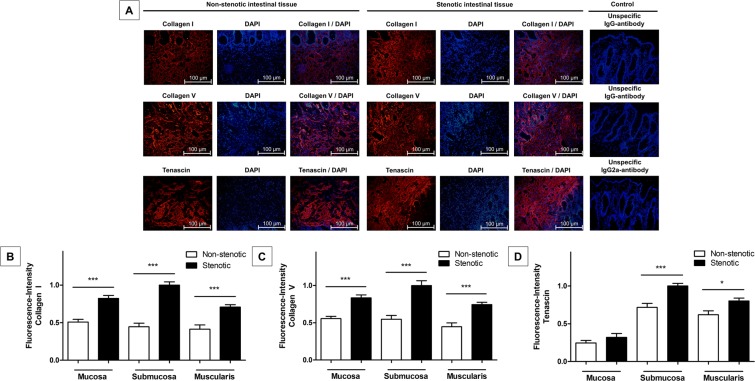
Immunofluorescence studies of non-stenotic and stenotic intestinal tissue of Crohn’s disease patients. (**A**) Cryostat sections of 30 surgical resection specimen of stenotic and non-stenotic intestinal tissue of 15 Crohn’s disease patients were stained with markers of fibrosis (Collagen I, Collagen V and Tenascin). Depicted is the submucosal tissue of non-stenotic (left) and stenotic bowel tissue (right). (**B–D**) Fibrosis markers were detectable in both, non-stenotic and stenotic bowel tissue; however, Collagen I, Collagen V and Tenascin were significantly elevated in stenotic compared to non-stenotic tissue in the “overall”- (all p < 0.001) and the “per-bowel-wall-layer”-analysis (all p < 0.02; except for mucosal Tenascin fluorescence intensity which did not significantly differed between stenotic and non-stenotic tissue). Data are mean ± standard error of mean (SEM). Statistical analysis was performed using Mann-Whitney U test. Two-sided p values < 0.05 were considered statistically significant.

To determine the degree of fibrosis in our tissue samples, immunofluorescence studies were performed using established fibrosis markers (collagen I, collagen V and tenascin)^23–25^. Collagen I, collagen V and tenascin were detectable in non-stenotic as well as stenotic parts of the bowel wall and were found in all layers of the bowel wall (Fig. 3). For all markers, the fluorescence intensity of fibrotic markers was significantly elevated in stenotic compared to non-stenotic tissue samples (all p < 0.001). In detail, differentiated by bowel wall layer, the fluorescence intensity of all fibrotic markers was significantly increased in all bowel wall layers in stenotic compared to non-stenotic tissue samples (all p < 0.02; except for the mucosal fluorescence intensity of tenascin which did not significantly differed between stenotic and non-stenotic tissue samples; Fig. 3B–D). This finding reflects a significantly advanced degree of fibrosis in all stenotic tissue samples and that fibrosis was detectable, but also to a lower extent, in non-stenotic tissue samples.

### Correlation between the refractive index and intestinal fibrosis

**Figure 4:**
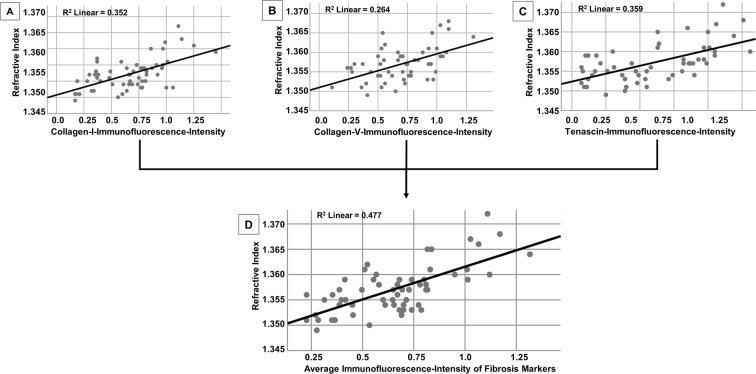
Significant correlation between the refractive index (RI) determined by digital holography microscopy (DHM) and the immunofluroescence-intensity of fibrosis markers. (**A–C**) The RI of the intestinal tissue (mucosa, submucosa and muscularis propria) determined by DHM in 30 surgical resection specimens significantly correlated with the light intensity of fibrosis markers determined by immunofluorescence studies (all p < 0.001). (**D**) The strongest correlation was found between the RI and the average immunofluorescence-intensity of all three fibrosis markers (p < 0.001; R^2^ = 0.477). Statistical analysis was performed using Pearson-correlation. Two-sided p values < 0.05 were considered statistically significant.

Next, we evaluated the correlation between the RI determined by DHM and the presence of fibrotic markers (collagen I, collagen V and tenascin) as reflected by immunofluorescence: for this analysis, the mean RIs and the mean fluorescence intensity signals of each bowel wall layer (mucosa, submucosa and muscularis propria), each determined in three images (digital holograms/immunofluorescence images, respectively) and evaluated separately according to surgical resection specimen, were correlated with each other. Overall, we found a strong correlation between the RI and the degree of all fibrotic markers (all p < 0.001; Fig. 4). More specifically, the RI correlated well with collagen I (R^2^ = 0.352, p < 0.001; Fig. 4A) and to a slightly lesser extent with collagen V (R^2^ = 0.264, p < 0.001, Fig. 4B) as well as with tenascin (R^2^ = 0.359, p < 0.001, Fig. 4C). All three markers account for fibrotic tissue alterations. Thus, the average of all three markers might be the most appropriate indicator to reflect the actual amount of fibrosis within the examined tissue. Confirmatively, the average fluorescence-intensity of all three fibrosis markers correlated best with the RI (R^2^ = 0.477, p < 0.001; Fig. 4D).

### Tissue stiffness is increased in fibrotic strictures

**Figure 5:**
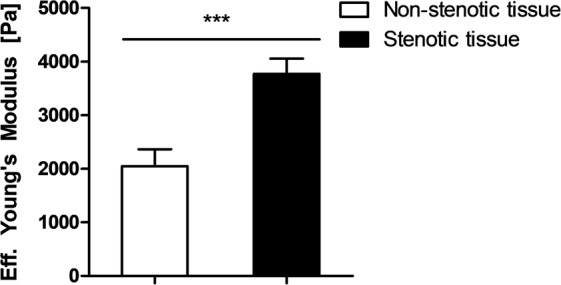
Tissue stiffness of stenotic intestinal tissue was significantly elevated compared to non-stenotic intestinal tissue of Crohn’s disease patients. Using a cantilever-based nanointender (*Piuma Nanoindenter* by Optics11, Amsterdam, N.L.), the Young’s Modulus of cryostat sections of non-stenotic and stenotic segments of the intestinal wall were assessed. Performing 139 measurements (n = 44 in non-stenotic tissue and n = 95 in stenotic tissue), stenotic tissue had a significant higher stiffness compared to non-stenotic tissue (p < 0.001). Pa = Pascal. Data are mean ± standard error of mean (SEM). Statistical analysis was performed using Mann-Whitney U test. Two-sided p values < 0.05 were considered statistically significant.

In addition to the immunofluorescence studies of fibrosis, tissue stiffness of the obtained surgical resection specimen of CD patients was analysed as a sign for organ fibrosis using the cantilever-based nanointender *(Piuma Nanoindenter*, Optics11, Amsterdam, N.L.). The Young’s Modulus was determined in all layers of the intestinal wall (mucosa, submucosa and muscularis propria): in total, 139 stiffness measurements were performed, subdivided into 44 stiffness measurements from non-stenotic tissue areas and 95 measurements from stenotic tissue areas, each obtained from one tissue sample. Confirming previous results, the stiffness of stenotic tissue was significantly elevated compared to tissue of non-stenotic parts of the bowel (3772 ± 282 Pa vs. 2048 ± 314 Pa; p < 0.001; Fig. 5).

## Discussion

Our study shows that QPI using DHM is feasible in accurately assessing fibrotic alterations in tissue samples of CD patients. Therefore, it might possess an additive value in the challenging characterization and thus more individualized therapy of CD-associated strictures in the future.

QPI using DHM offers several unique features: it operates label-free^17,18,26^ and due to its interferometry-based concept to measure the OPLD, it allows a highly accurate quantification of tissue density and requires only minimized calibration and sample preparation demands^9,18,20,21^. We previously demonstrated DHM to be capable of accurately detecting inflammatory mediated single cell alterations as well as colonic changes^16,18,19,22^. In DSS-treated colitic mice, the RI was significantly decreased in all layers of the colonic wall as compared to healthy controls^18^. In line with these findings, RI values were significantly decreased in colonic biopsies from human CD patients with an acute flare compared to CD patients in remission^19^. Additionally, with tissue analysis, DHM examination of intestinal epithelial Caco-2 cells during wound closure experiments *in-vitro* allowed the determination of cell proliferation and migration. DHM provided cellular parameters of these wounded cells including volume, density, thickness and dry mass over the course of time^22^. Apart from IBD, DHM was feasible in monitoring biophysical cell properties of C6/36 cells during dengue virus infection and characterized morphological alterations of erythrocytes during Malaria infection^27,28^.

To the best of our knowledge, our study is the first to evaluate DHM in quantifying fibrotic tissue alterations in human samples. Based on our findings, DHM was able to differentiate between fibrotic and non-fibrotic tissue areas. Of note is the fact that no fully validated histopathological scoring system to evaluate fibrosis in CD is available to date^16,29^. While some scoring systems applied only a rather crude fibrosis classification^30,31^, others do not include the analysis of fibrosis severity for each bowel wall layer separately^32^. In CD patients, fibrosis occurs in all layers of the bowel wall leading to a transmural thickening and stiffening accompanied by changes of the extracellular matrix (ECM)^5,33^. The ECM is primarily formed by different collagen subtypes deposited within the submucosal layer and muscularis propria^5,23,33^. Our results show a good correlation of the RI value with collagen I and V^5,23,33^ and the ECM protein tenascin, which has been previously shown to be present in stricturing CD^24^. In our hands, fibrosis markers were not only present in stenotic, but also to a significantly lower extent in non-stenotic tissue samples. This may be partially explained by the fact that the non-stenotic tissue samples were obtained from intestinal tissue adjacently localized to the stricture. Interestingly, we observed the best correlation between RI values and the average fluorescence intensity of all three included fibrosis markers. It might be speculated, that the average fluorescence intensity of all three fibrosis markers best represents the degree of tissue fibrosis, because it might most appropriately considers local distribution differences of ECM proteins^23,24^.

An increased stiffness is generally observed in fibrotic organs and caused by the deposition of ECM proteins like collagen and their cross-linking^34^. Accordingly, diagnostic assessment of organ stiffness by US elastography has been established in the clinical management of patients with liver fibrosis^27,35^. Recently, a few pilot studies evaluated US elastography for the assessment of stricturing CD^28,36,37^; however, its’ spatial resolution is poor and this technique is currently not recommended by international guidelines^5,38^. The improvement of current diagnostics using assessment of tissue elasticity by microscope placement might contribute to the characterization of stricturing CD. Moreover, recently, optical coherence elastography (OCE) has been introduced in experimental studies: OCE operates at a microscopic scale and outperforms US elastography in terms of spatial resolution^38,39^. We utilized a commercially available cantilever-based nanoindenter instrument which comparably operates on a microscopic scale^40,41^. Due to the timely restricted availability of this nanoindenter instrument in our study, we were only able to perform a limited number of elasticity measurements. However, in these preliminary proof of concept experiments, we detected a significant higher stiffness in stenotic compared to non-stenotic tissue which further supports our previous histopathological and immunofluorescence findings and indicates a higher fibrotic content in stenosed tissue areas.

Recently, consensus recommendations for the evaluation, interpretation and utilization of CT and MRI in IBD patients were published with the conclusion that no current imaging modality accurately estimates the degree of fibrosis within CD strictures and likewise no current imaging modality is validated for fibrosis assessment^42^. For instance, Adler *et al*. retrospectively evaluated CT for the assessment of CD strictures and successfully differentiated inflammation and fibrosis in subgrades; however, they applied no safe differentiation between inflammatory and fibrotic tissue alterations^43^. Furthermore, Wilkens *et al*. recently employed contrast-enhanced ultrasound (CEUS) and contrast-enhanced (CE) MRI for the assessment of small bowel CD strictures and found no correlation between the severity of inflammation nor fibrosis on histopathology and CEUS nor CE-MRI^44^. These results indicate the limitation of current imaging modalities to sufficiently determine the degree of fibrosis within CD strictures. Nevertheless, there is great unmet need to determine fibrotic components in CD strictures, especially given the fact that clinical trials evaluating anti-fibrotics in patients with stricturing CD are foreseeable^45^.

In light of our results, DHM might contribute to accurately assessing fibrosis within CD strictures in the future. Due to its operation principle to determine the OPLD, DHM offers absolute values for tissue density^18,20,21^. Taking this into account, repetitive tissue density measurements during the patient’s course of disease might provide important information for therapeutic monitoring and furthermore these absolute values might make results of clinical trials easily comparable.

Our study might have several limitations: first, this study included only a limited number of patients and tissue samples; however, this is a proof of concept study demonstrating the use of DHM for CD stricture assessment for the first time. Secondly, the overall RI determined by DHM was significantly higher in stenotic compared to non-stenotic tissue, but the detailed analysis only revealed significant differences in the submucosal layer and not in the mucosa and the muscularis propria. This finding might be explained by the limited number of patients and tissue samples, the predominance of collagen deposition in the submucosa of CD strictures^23^ and furthermore, our control samples were obtained from the intestine located adjacently to the stricture and therefore may also incorporated fibrotic tissue alterations which is validated by the results of the correlative study with immune fluorescence microscopy. Thirdly, fibrosis assessment using DHM might not be superior to the detailed histological analysis of tissue; however, DHM appears to be a promising tool with future prospects of acquiring reliable supporting objective and quantitative biophysical tissue data. Recently, experimental studies were reported in which endoscopes were equipped with optical coherence tomography (OCT): OCT provides high resolution images of tissue microstructures deep into the tissue by measuring back-scattered or back-reflected light using interferometric phase information^46–48^. Thus, complementary RI and tissue density data as provided by QPI with DHM can be expected to contribute significantly to the interpretation of the diagnostic findings retrieved by recently developed and commercialized endoscopic OCT systems^46–49^. On the other hand, in future, the comparison of DHM data obtained from correlated studies with endoscopic OCT could be used to validate our findings and promises a further advanced characterization of CD strictures. Fourth, CD strictures frequently consist of inflammatory and fibrotic tissue compounds^50,51^. In this study, patients with a predominant fibrotic stricture phenotype were included and considering our results, DHM is feasible to accurately assess the degree of fibrosis within CD strictures; however, also the differentiation of predominant fibrotic from predominant inflammatory strictures appears to be an important aspect that should be addressed in future studies on DHM-based stricture assessment with the aim to identify RI values, which could indicate predominant fibrotic strictures warranting primary surgical intervention.

Taken together, our data indicate that DHM as an example of QPI, may accurately assess fibrotic alterations in tissue samples of CD patients and result in characterizing CD strictures in terms of digital histopathology in the future.

## Methods

### Study design and inclusion criteria

This proof of concept study was performed at the Department of Medicine B for Gastroenterology and Hepatology and the Department of General and Visceral Surgery of the University Hospital Muenster and at the Biomedical Technology Center, University of Muenster, Muenster, Germany. The study was approved by the Ethics Board of the University of Muenster and the Medical Council of Westphalia-Lippe, Germany and conforms to the ethical guidelines of the 1975 Declaration of Helsinki. After informed consent was obtained, data and tissue samples from patients ≥18 years of age who underwent a surgical resection of a CD-associated intestinal stricture were included in this study. Disease characteristics of included patients are shown in Table 1.

### Histological evaluation

From each patient a full thickness surgical resection specimen of the stenotic and of the adjacent, non-stenotic bowel section was obtained. The surgical resection specimens were embedded in O.C.T. (Optimal Cutting Temperature – Tissue Tek, Sukura Fine Tek Europe, Zoeterwoude, N.L.) and kept frozen at −80 °C until further use. Cryostat sections (7 µm) were stained with H&E and the presence of stenotic, predominant fibrotic tissue and non-stenotic tissue was confirmed by an expert pathologist (M.V.) with attention to changes in the crypt architecture and the amount of submucosal fibrosis.

### Quantitative phase imaging with digital holographic microscopy

For DHM analysis, an inverted microscope (iMIC, Till Photonics, Gräfelfing, Germany) with an attached digital holographic microscopy module was used (for further details, see Kemper *et al.*^19,52^). The applied light source was a frequency doubled neodymium-doped yttrium aluminium garnet (Nd:YAG) laser (Compass 315M-100, Coherent, Lübeck, Germany, λ = 532 nm). For investigation, the cryostat sections were placed on object glass carrier slides, embedded in phosphate buffered saline (PBS), and covered with a coverslip. Digital off-axis holograms of crystat sections were recorded with a charge coupled device sensor (DMK 41BF02, The Imaging Source GmbH, Bremen, Germany) using a 10x microscope lens (Zeiss EC Plan-Neoflura 10 × 0.3, NA = 0.3) and numerically reconstructed utilizing spatial phase shifting in combination with optional holographic autofocusing as described previously^18,19^. The resulting quantitative phase images were used to quantify the OPLD caused by the investigated cryostat sections of intestinal tissue samples.

### Determination of the refractive index using digital holographic microscopy

The RI reflects the cell and tissue density^14,16–18^: For single cells, the RI quantifies the concentration of intracellular solutes such as osmotic active compounds like proteins^53–55^, while in the analysis of tissue samples, it includes data on the intracellular content and also on the contribution from extracellular compounds such as matrix proteins^18,19^. For mainly transparent specimens, such as the investigated intestinal cryostat sections, with a constant thickness *d*_*s*_, a spatial varying integral *RI*_*s*_*(x, y)* in a surrounding medium with a constant refractive index *RI*_*medium*_ and a constant wave length λ of the laser light source utilized for DHM, the OPLD phase change *Δφ(x, y)* to the surrounding medium is:

1 $$\varDelta \varphi (x,y)=(\frac{2\pi }{\lambda })\ast {d}_{s}\ast (R{I}_{s}(x,y)\,-R{I}_{medium})$$

Thus, from Eq. 1, for a tissue sample with a constant thickness *d*_*s*_ and available parameters for *RI*_*medium*_ and *λ* (here: *d*_*s*_ = *7 µm, RI*_*medium*_ = *RI*_*PBS*_ = *1.337 and λ* = *532* *nm*), the spatial distribution of RI_*s*_*(x, y)* can be calculated:

2 $$R{I}_{s}(x,y)=(\frac{\varDelta \varphi s(x,y)\ast \lambda }{d\ast 2\pi })+R{I}_{medium}$$

### Evaluation of density changes of intestinal tissue of CD patients by digital holographic microscopy

Cryostat sections of full thickness surgical resection specimen (n = 30) of non-stenotic (n = 15) and stenotic (n = 15) parts of the intestinal wall from the same patient, obtained from 15 CD patients with a symptomatic intestinal stricture and the need for a surgical resection, were analyzed using DHM (Fig. 1). The quantitative DHM phase contrast images, retrieved by the numerical reconstruction of the digital holograms as described in section “Quantitative phase imaging with digital holographic microscopy” were evaluated using the public image processing and analysis software ImageJ version 1.45 (NIH, Bethesda, MD, U.S.)^56^. Bright regions in quantitative phase images reflect a higher OPLD resulting in a higher RI, which correlates to a high tissue density; likewise, the darker regions represent a lower OPLD and correspond to areas with a lower tissue density. In order to quantify the tissue density systematically, segmental analysis of the tissue was performed according to the layered structure of the intestinal wall (detailed described and depicted in Lenz *et al*. 2013, Fig. 1)^18^. For each wall layer (mucosa, submucosa and muscularis propria) of an analyzed specimen, three digital holograms were acquired, finally resulting in nine digital holograms recorded in different fields of view that were analyzed from each tissue sample. The RI was assessed in each of the corresponding quantitative images in an appropriate, sized-defined region of interest. Note that due to the changing quality of the surgical resection specimen, for example, caused by severe fibrotic alterations within the CD stricture, in some of the samples not all three layers of the intestinal wall were preserved.

### Immunofluorescence studies for the assessment of fibrosis in intestinal tissue

Cryostat sections of 30 surgical resection specimens of the intestinal wall (thickness = 7 µm; 15 specimens of stenotic and non-stenotic bowel parts each) were air-dried, fixed for 10 minutes in pure acetone at −20 °C and blocked with blocking buffer (PBS containing 10% goat serum) for 60 minutes at room temperature. A two-step staining was applied: first, slides were incubated with solutions of (a) a purified polyclonal rabbit anti-collagen I antibody (dilution 1:1000 in PBS with 0.5% BSA, abcam, Cambridge, U.K.), (b) a purified polyclonal rabbit anti-collagen V antibody (dilution 1:1000 in PBS with 0.5% BSA, abcam, Cambridge, United Kingdom) or (c) a purified monoclonal mouse anti-tenascin antibody (dilution 1:1000 in PBS with 0.5% BSA, abcam, Cambridge, United Kingdom) and second, slides were incubated with the secondary antibody (anti-rabbit IgG Alexa Fluor 546 or anti-mouse IgG Alexa Fluor 546; both dilution 1:1500, Invitrogen – Thermo Fisher, Waltham, United States). Additionally, cell nuclei were counterstained with DAPI (4,6-diamidino-2-phenylindole dihydrochloride, Invitrogen - Thermo Fisher, Waltham, United States). Images were acquired using a fluorescence microscope with a 20x microscope lens (Leica DMBL, Leica Microsystems, Wetzlar, Germany).

For the systematic analysis of tissue samples, similar to the investigations with quantitative DHM phase contrast, a segmental analysis of the tissue was performed according to the layered structure of the intestinal wall (mucosa, submucosa and muscularis propria). For each wall layer and fibrosis marker (collagen I, collagen V and tenascin), three fluorescence images were taken with the same microscope settings. Due to severe fibrotic alterations within the CD stricture, in some of the samples not all three layers of the intestinal wall were preserved.

The fluorescence intensity of each bowel wall layer was assessed choosing an appropriate, sized-normed region of interest in each fluorescence image using ImageJ version 1.45 (NIH, Bethesda, MD, U.S.)^56^.

### Assessment of the tissue stiffness of intestinal tissue

For correlative tissue stiffness assessment, cryostat sections with a thickness = 30 µm were cut from both, stenotic and non-stenotic intestinal tissue samples, placed on microscope glass carrier slides and embedded in PBS. A cantilever-based nanointender (*Piuma Nanoindenter*, Optics11, Amsterdam, N.L.), which utilizes fiber-based optical interferometry to measure the indentation force and displacement at nanometer level, was applied. The instrument allows the determination of the Young’s Modulus in the range from 5 pascal (Pa) up to 1 gigapascal (GPa), utilizing a spherical indentation tip (stiffness k = 0.04 N/m, radius of the sphere at the tip: 61 µm). After positioning the indenter probe slightly above the surface of the sample, it applied a force to the sample and the load and surface displacement were recorded which allowed the determination of the Young’s Modulus in a subsequent evaluation^40^.

### Statistical analysis

Mean and SEM were determined for continuous variables, and frequencies and percentages are provided for categorical variables. Statistical data analysis was performed using IBM SPSS Statistics 25.0 (IBM Corp., Armonk, U.S.) utilizing the Mann-Whitney U test and Pearson correlation. Two-sided *p* values < 0.05 were considered to be statistically significant.

## Supplementary information

Supplementary Information

## Data Availability

All data generated or analysed during this study are included in this article. The original datasets are available from the corresponding author on reasonable request.
