# Supplementary material for: Quantitative Phase Imaging Using Digital Holographic Microscopy Reliably Assesses Morphology and Reflects Elastic Properties of Fibrotic Intestinal Tissue
Source: Sci Rep. 2019 Dec 18;9:19388. doi: 10.1038/s41598-019-56045-2 (PMC6920451; doi:10.1038/s41598-019-56045-2)
Supplement: Supplementary file 1 — Supplementary Information [file 41598_2019_56045_MOESM1_ESM.pdf]

QUANTITATIVE PHASE IMAGING USING DIGITAL HOLOGRAPHIC MICROSCOPY RELIABLY ASSESSES MORPHOLOGY AND REFLECTS ELASTIC PROPERTIES OF FIBROTIC INTESTINAL TISSUE

Arne Bokemeyer[1], Phil Robin Tepasse[1], Lena Quill[1], Philipp Lenz[2], Emile Rijcken[3], Michael Vieth[4], Nik Ding[5], Steffi Ketelhut[6], Florian Rieder[7], Björn Kemper[6*], Dominik Bettenworth[1*]

*Authors contributed equally and are both considered last authors

[1]Department of Medicine B for Gastroenterology and Hepatology, [2]Institute of Palliative Care, [3]Department of General, Visceral and Transplantation Surgery, University Hospital Muenster, Germany. [4]Institute of Pathology, Klinikum Bayreuth, University of Erlangen-Nuremberg, Bayreuth, Germany. [5]Department of Medicine, University of Melbourne, Melbourne, Vic, Australia. [6]Biomedical Technology Center, University of Muenster, Muenster, Germany. [7]Department of Gastroenterology, Hepatology and Nutrition, Digestive Diseases and Surgery Institute, Cleveland Clinic, Cleveland, Ohio, USA. *Correspondence and request for materials should be addressed to B.K. (email: bkemper@uni-muenster.de)

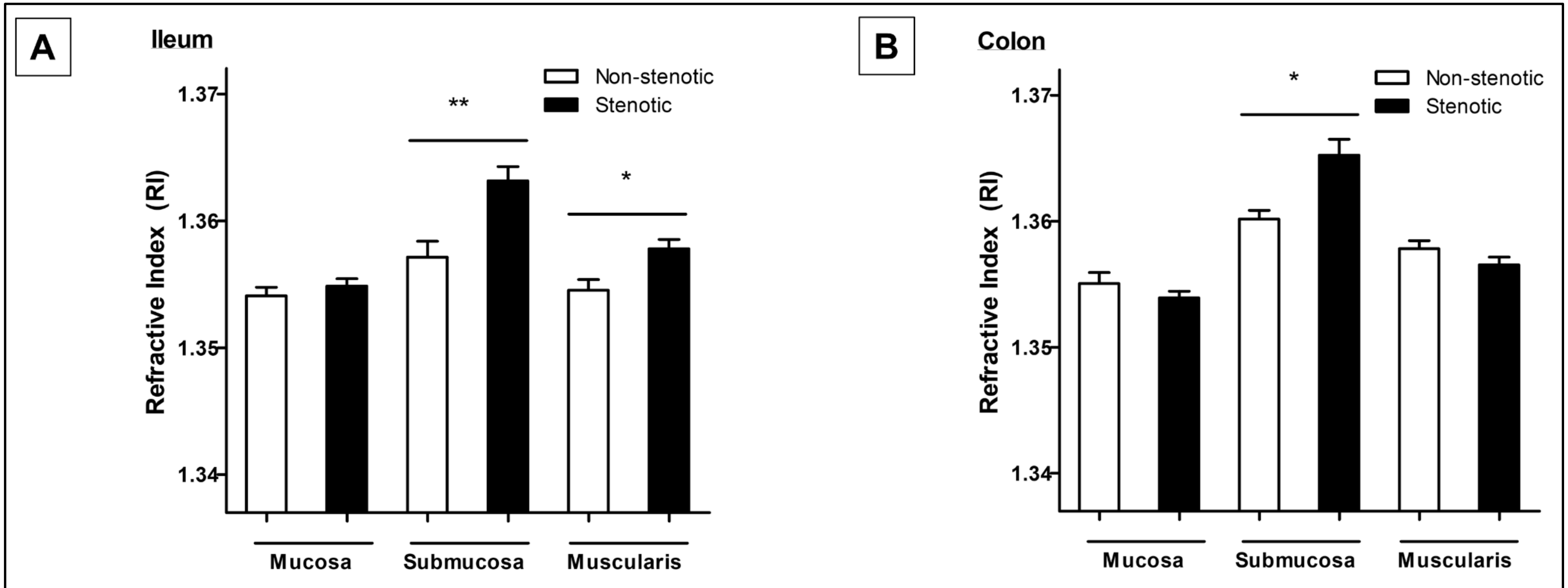

Type of tissue	Bowel wall layer	Non-stenotic tissue	Stenotic tissue	P value
		RI	RI	
**Ileum (n=10)**	**Mucosa**	1.354±0.001	1.355±0.001	0.585
	**Sub-mucosa**	1.357±0.001	1.363±0.001	**0.004****
	**Mus-cularis**	1.355±0.001	1.358±0.001	**0.017***

Type of tissue	Bowel wall layer	Non-stenotic tissue	Stenotic tissue	P value
		RI	RI	
**Colon (n=5)**	**Mucosa**	1.355±0.001	1.354±0.001	0.505
	**Sub-mucosa**	1.360±0.001	1.365±0.001	**0.01***
	**Mus-cularis**	1.358±0.001	1.357±0.001	0.176

Supplementary Information: Determination of the refractive index of small bowel and large bowel tissue of Crohn‘s disease (CD) patients using digital holographic microscopy (DHM). In total, 20 small bowel surgical resection specimen from 10/15 CD patients and 10 large bowel surgical resection specimen from 5/15 CD patients were evaluated. The refractive index (RI), determined by DHM, was assessed in all layers of the bowel wall (mucosa, submucosa, muscularis propria). The RI was significantly elevated in stenotic tissue samples of the submucosa in both, ileum and colonic tissue (p_{Ileum}=0.004 and p_{Colon}=0.01). Data are mean ± standard error of mean (SEM). Statistical analysis was performed using Mann-Whitney U test. Two-sided p values < 0.05 were considered statistically significant. Abbreviations: CD, Crohn’s Disease; DHM, digital holographic microscopy; RI, refractive index; SEM, standard error of mean.
